# Design of a Capacitive Tactile Sensor Array System for Human–Computer Interaction

**DOI:** 10.3390/s24206629

**Published:** 2024-10-14

**Authors:** Fei Fei, Zhenkun Jia, Changcheng Wu, Xiong Lu, Zhi Li

**Affiliations:** 1 College of Automation Engineering, Nanjing University of Aeronautics and Astronautics, Nanjing 211100, China; jzk@nuaa.edu.cn (Z.J.); changchengwu@nuaa.edu.cn (C.W.); luxiong@nuaa.edu.cn (X.L.); 2 College of Computer Science and Software Engineering, Shenzhen University, Shenzhen 518060, China

**Keywords:** capacitive sensing, tactile sensor, inkjet deposition printing, human–computer interaction

## Abstract

This paper introduces a novel capacitive sensor array designed for tactile perception applications. Utilizing an all-in-one inkjet deposition printing process, the sensor array exhibited exceptional flexibility and accuracy. With a resolution of up to 32.7 dpi, the sensor array was capable of capturing the fine details of touch inputs, making it suitable for applications requiring high spatial resolution. The design incorporates two multiplexers to achieve a scanning rate of 100 Hz, ensuring the rapid and responsive data acquisition that is essential for real-time feedback in interactive applications, such as gesture recognition and haptic interfaces. To evaluate the performance of the capacitive sensor array, an experiment that involved handwritten number recognition was conducted. The results demonstrated that the sensor accurately captured fingertip inputs with a high precision. When combined with an Auxiliary Classifier Generative Adversarial Network (ACGAN) algorithm, the sensor system achieved a recognition accuracy of 98% for various handwritten numbers from “0” to “9”. These results show the potential of the capacitive sensor array for advanced human–computer interaction applications.

## 1. Introduction

The capacitive tactile sensor is a type of sensor based on the principle of capacitance variation, which is used to simulate human tactile perception. It can measure the contact pressure and position between an object and the sensor and convert them into electrical signals. The working principle of a capacitive tactile sensor is based on changes in capacitance. When an object comes into contact with the surface of the sensor, a capacitive effect is generated. Specifically, the sensor consists of two electrodes, one fixed and the other movable. When an object touches the sensor, a capacitance is formed between the object and the electrode, and the size of this capacitance depends on the contact area and pressure of the object. By measuring the change in capacitance, the sensor can determine the contact position and pressure of the object. Capacitive tactile sensors have several essential characteristics. First, they can provide high-resolution tactile feedback. Due to the sensitivity of capacitance changes in response to even slight variations in the contact pressure, capacitive tactile sensors can provide delicate tactile information. Second, capacitive tactile sensors have fast response times. They can detect and measure the contact status of an object in real time, allowing for quick feedback to the system or user. Additionally, capacitive tactile sensors can achieve multi-touch and multi-dimensional touch functionality, making them widely applicable in interactive devices and virtual reality.

Capacitive tactile sensors can be categorized into two types based on their sensing principles. One type is the parallel plate capacitor, which relies primarily on changes in the distance between two electrodes or variations in the dielectric constant of the dielectric layer when an external object comes into contact, resulting in changes in the capacitance for detecting external touches. Fu et al. introduced a cantilever-based single-crystal diamond capacitive pressure sensor designed for harsh environments with high temperatures, oxidation, high radiation, and strong corrosion [1]. He et al. developed an array-type capacitive sensor with a sandwich-like structure using a flexible, low-cost nylon netting as the inter-electrode material, achieving high responsiveness and stable operation [2]. Kou et al. employed a graphene/polydimethylsiloxane (GR/PDMS) sponge as the dielectric layer, along with copper electrodes featuring distinct patterns and an antenna, to create a flexible wireless pressure capacitive sensor for monitoring finger bending and facial muscle movements [3]. Atalay et al. designed a capacitive flexible pressure sensor utilizing conductive fabric as parallel plate electrodes and a silicone elastomer as the dielectric layer, achieving exceptionally high sensitivity. The capacitive sensor was integrated into a textile glove to monitor grasp motions during daily activities [4]. To further enhance the sensitivity of capacitive pressure sensors, Ruth et al. proposed an improved manufacturing method incorporating a pyramid microstructured dielectric layer, along with a lamination layer, resulting in tunable, consistent, and reproducible pressure sensors [5]. Addressing the low transparency issue of traditional porous materials or microstructured dielectric layers, Liu et al. filled a porous polyvinylidene fluoride (PVDF) film with an ionic liquid, obtaining a capacitive sensor with high transmittance [6].

Another type of capacitive tactile sensor is the proximity capacitive sensor, which detects external stimuli by observing variations in capacitance resulting from changes in the surrounding dielectric constant when an object approaches. In contrast to capacitive sensors relying on their deformation for pressure sensing, proximity capacitive sensors typically feature interleaved electrode arrangements. Due to the lack of necessity for a specifically designed dielectric layer, these sensors exhibit a lighter and thinner profile and can detect objects at greater distances. Huang et al. developed a capacitive proximity sensor by utilizing a new type of complementary Archimedean spiral electrode [7]. Zhao et al. designed a fully paper-based capacitive pressure and proximity sensor with a customizable shape by employing multi-layer tissue paper as the dielectric and polypyrrole-coated paper as the electrode [8]. Tabrizi et al. proposed a fully integrated complementary metal–oxide–semiconductor (CMOS) 16 × 16 capacitive sensor array designed for life science applications; this sensor was applied in the analysis of various chemical solvents [9]. Kong et al. manufactured a low-cost, novel flexible capacitive pressure-pulse sensor array using graphene oxide for human wrist pulse sensing. Yang et al. proposed a flexible pressure sensor based on double-layer capacitors between ITO-PET electrodes and a PVA/KOH membrane [10]. Saqib et al. utilized a natural inner eggshell membrane (IESM) to create a biocompatible, capacitive, and self-powered piezoelectric pressure sensor array for detecting static and dynamic pressures [11]. Wang et al. devised a flexible wearable hand gesture recognition system based on a capacitive pressure sensing array and deep convolutional neural networks. The capacitive array integrated into a flexible wristband is proficient at recognizing various hand gestures [12].

In this study, high-resolution capacitive tactile sensors were fabricated using inkjet deposition printing technology. Their remarkable lightweight features render them applicable in the wearable area. A computer program for capacitance measurement and graphical display was created, allowing users to draw numbers on the sensor surface and compare them with measured capacitance values to verify the precision of touch point detection. Furthermore, a series of handwritten number recognition experiments based on the ACGAN algorithm was designed to evaluate the potential of the sensor in human–computer interaction.

## 2. Sensor Fabrication

The capacitive sensor device designed in this study consists of two main components: (1) a sensing area that captures the touching distribution, and (2) a circuit that controls the sensing elements and transmits the data to the terminal for graphical data analysis. The sensing area is based on the principle of mutual capacitance. It comprises two layers of overlapping conductors: one contains multiple transmitting electrodes. In contrast, the other layer contains multiple receiving electrodes, with the two layers electrically insulated from each other by a dielectric material [13]. The transmitting and receiving electrodes are arranged in a 2D matrix pattern, resulting in overlapping intersection points. Each intersection point is formed by a pair of transmitting and receiving electrodes, creating micro-capacitor units that exhibit mutual capacitance. The capacitance value of each micro-capacitor unit varies according to Equation (1).

(1) $$C_{sensor}=\varepsilon_{o}\varepsilon_{r}\cdot A/d_{0}$$

In Equation (1), $C_{sensor}$ represents the capacitance value of the micro-capacitor, $\varepsilon_{o}$ is the permittivity constant in a vacuum, $\varepsilon_{r}$ signifies the dielectric constant of the material, A denotes the overlapping area between the two electrodes, and $d_{0}$ refers to the distance separating the electrodes. When a person’s finger approaches and touches one of the intersection points, it disrupts the electric field between the two electrodes, resulting in a change in the capacitance value at that point [14]. By employing time-division multiplexing, the capacitance values at each intersection point in the array can be scanned and measured, allowing for the capacitance distribution across the surface of the capacitive sensor to be obtained.

**Figure 1:**
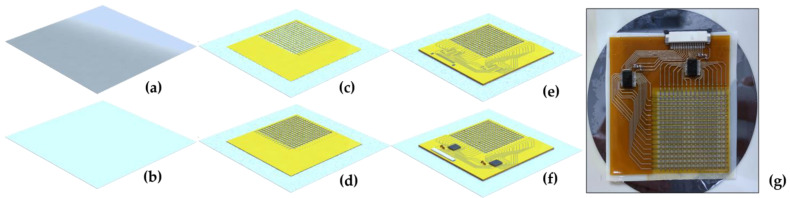
Sensor fabrication process: (**a**) a polyester film as a printing substrate; (**b**) PVA coating onto the polyester film; (**c**) printing process of the row electrode; (**d**) printing process of the column electrode; (**e**) printing process of the interconnects; (**f**) soldering of electronic components, and (**g**) the final fabricated sensor device.

The fabrication approach for realizing the thin and flexible capacitive sensors is demonstrated in this paper. To realize the printing process for this tactile sensor device, an inkjet deposition printer is required [15]. The DragonFly LDM system from Nano Dimension Ltd. was utilized to integrate both the sensor and circuit design in one printing process. The detailed fabrication process is shown in Figure 1.

First, a polyester film in Figure 1a as a printing substrate was coated with a 100 µm water-soluble synthetic polymer in Figure 1b, namely, polyvinyl alcohol (PVA), which was used for detaching the printed sensor from the substrate after the printing process. Second, the coated substrate was placed in the printing platform, and the printing process was subsequently started to convert the designed 3D model into slices. Third, the dielectric and conductive inks were printed layer-by-layer according to the slicing result, and the printing process was completed after 9 h, as shown in Figure 1c–e. Fourth, the printed sensor was soldered with the required electronic components (capacitors, resistors, connectors, and multiplexers) in Figure 1f. Fifth, the soldered sensor was spin-coated with 100 µm PDMS, then baked in an oven at 60 °C for 1 h. Lastly, the sensor was submerged in deionized water for 6 h to detach it from the polyester substrate.

Following the demonstrated fabrication process, a 16 × 16 flexible capacitive tactile sensor was successfully obtained, as shown in Figure 1g. The sensor’s dimensions were 70 mm × 70 mm × 1 mm, and the sensing area covered 40 mm × 40 mm, which resulted in an approximate resolution of 10.2 dpi. To facilitate the scanning and measurement of the entire sensor array, two multiplexers were thoughtfully affixed along the periphery of the sensing area, and they operated using a 5 V DC power supply.

**Figure 2:**
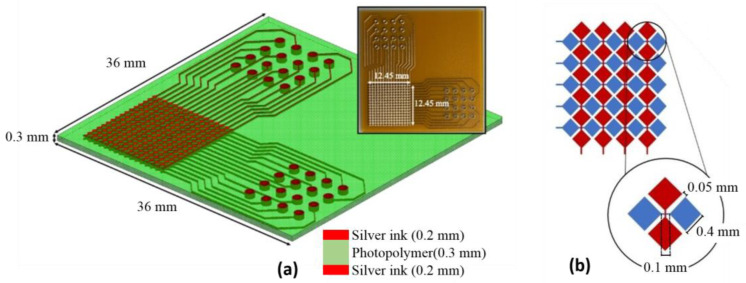
Design of the sensor system. (**a**) A 16 × 16 capacitive sensor with a pixel resolution of 32.7 dpi. (**b**) A 0.4 mm × 0.4 mm diamond sensing element and interconnects of 0.1 mm.

In order to maximize the exposure of sensor electrodes to a tactile change, which can provide a better sensing resolution and miniaturize the overall sensor size, the sensing area in Figure 2 was replaced with diamond-shaped elements using a similar fabrication process as in Figure 1. A 16 × 16 pixelated sensor with a resolution of 32.7 dpi is shown in Figure 2a. The sensor comprised a three-layer structure. The top layer was an electrode pattern made from 0.2 mm thick silver ink, the middle layer was a 0.3 mm thick photopolymer, and the bottom layer was another electrode pattern made from 0.2 mm thick silver ink. The electrode dimensions with exact spacing parameters are shown in Figure 2b. The size of the diamond-shaped element was 0.4 mm × 0.4 mm, and the interconnection between the two elements was 0.1 mm.

## 3. Design of Data Acquisition System

**Figure 3:**
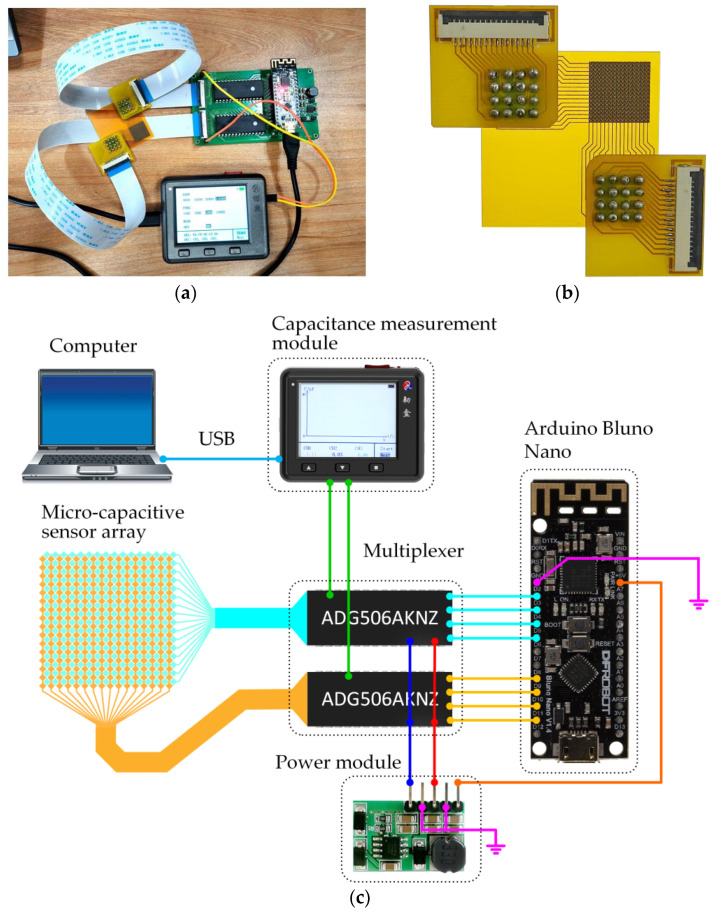
Demonstration of the capacitive tactical sensor system. (**a**) The hardware system includes the capacitive sensor, the Arduino controller, the capacitance measurement module, and two multiplexers. (**b**) High-resolution micro-capacitive array. (**c**) Connection of the hardware system.

To ensure a more flexible and scalable hardware system for the wearable sensor application, ribbon cables were used to connect the capacitive sensor to the control circuit [16]. A time-division multiplexing approach was implemented in this study to measure the capacitance distribution across the sensor accurately. Scanning the 256 micro-capacitors sensing elements was performed by controlling two eight-channel ADG506AKNZ multiplexers (one for rows and one for columns) using an Arduino Nano controller. The scanning frequency was set at 100 Hz. Moreover, a portable capacitance measurement module with an LCD display was integrated into the sensor system to measure the currently selected micro-capacitor and transmit the measured capacitance to the terminal for the data analysis. The overview and physical connection of the system are shown in Figure 3.

Having introduced the individual hardware components of the high-resolution capacitive sensor array, the next step involved connecting these components. The interconnection process is outlined below:

(1) The Arduino controller and the capacitance measurement device were connected to the computer via USB cables. This setup allowed for data transfer and control signals between the PC and the hardware components.
(2) The D2~D5 pins of the Arduino controller were connected to the four binary control pins of one analog switch. The D8~D11 pins of the Arduino controller were connected to the four binary control pins of another analog switch. It became possible to enable control over the input signals.
(3) The two inputs of the capacitance measurement module were connected to the outputs of both analog switches. The micro-capacitive array was connected to the 16 input channels of the two analog switches using flexible ribbon cables.

The hardware system was fully interconnected and ready for operation after completing these steps. This setup enabled the system to accurately measure and process the capacitance changes from the micro-capacitive array, which is essential for tactile perception applications.

## 4. Sensor Data Processing

External disturbances can introduce outliers in the measured capacitance values when acquiring capacitive tactile data using this sensor system. These anomalies can significantly affect the subsequent feature extraction processes and, consequently, degrade the overall performance. To mitigate these issues, pre-processing of the raw capacitance value sequences is necessary before performing visualization or further analysis. In the experiment, a 16 × 16 electrode array that consisted of 256 sensing elements was employed. In the absence of touch, multiple traversals were conducted across all elements, and the average value for each element was calculated as its inherent capacitance. This procedure was used to eliminate the influence of the inherent capacitance on the experimental touching values. The inherent capacitance of each element was approximately 9 pF.

### 4.1. Pre-Processing

(1) Data alignment:

In the capacitive tactile sensor system, the external capacitance measurement device controls the acquisition of capacitance values from the micro-capacitive array sensor surface and automatically sends the data to the computer. To ensure that the capacitance values received by the computer correspond to the correct positions on the sensor surface, it is necessary to align the capacitance data based on the timing of the channel switching controlled by the Arduino controller.

To synchronize the data acquisition process, the Arduino controller sends a channel-switching signal through the serial port when it controls the channel. This signal indicates that a channel-switching event has occurred. The channel-switching signal is “1” when the measurement begins for a new row and column (i.e., when the first row and first column of the micro-capacitive array are selected). For all other channel-switching events, the signal is “0”. The computer uses the timestamps of the received signals to align the capacitance values with the correct positions on the sensor surface. After the alignment, the data form a sequence containing 256 data points, corresponding to one complete traversal of the 16 × 16 micro-capacitive array. This aligned data sequence represents the capacitance values at each position of the sensor array for a single measurement cycle.

**Figure 4:**
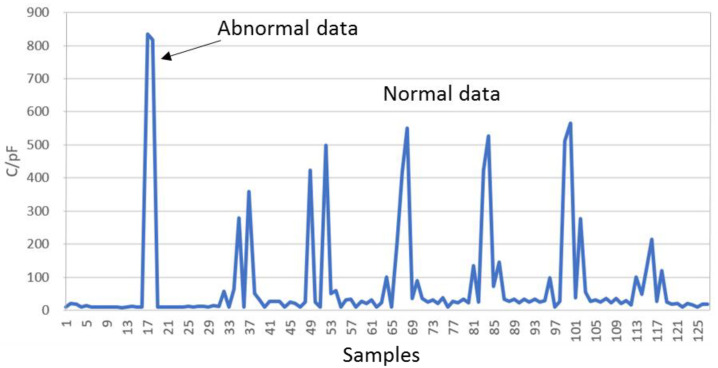
Comparison of abnormal and normal capacitance values.

(2) Abnormal data detection:

Due to external disturbances, occasional anomalies may occur in the capacitance value data. These abnormal data typically manifest as one or two exceptionally high capacitance values in non-contact regions. Figure 4 provides an excerpt of a capacitance value sequence showing normal and anomalous values. As illustrated, anomalous data often appeared as isolated peaks more significant than 800 pF, while normal data typically exhibited peaks around 500 pF, depending on the touching motion of the finger. Additionally, normal data tended to cluster together. Based on these characteristics, anomalous values could be identified and handled appropriately.

### 4.2. Acquisition of Capacitance Value Sequences

**Figure 5:**
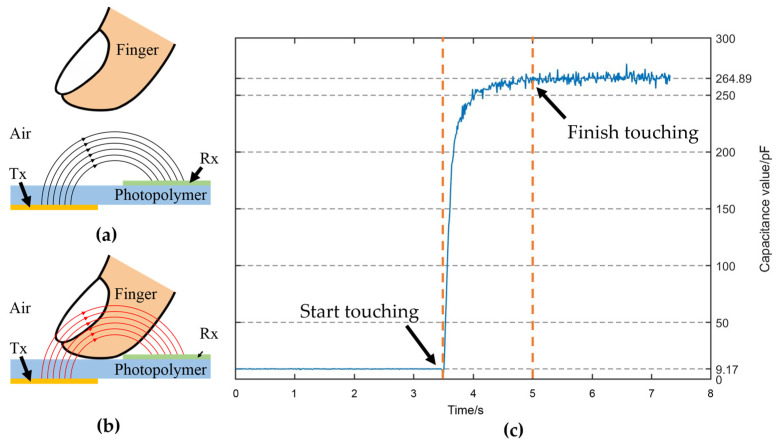
(**a**) The dielectric between the emitter and the receiver is the photopolymer and air without the finger touching. (**b**) The dielectric between the emitter and the receiver is the photopolymer, air, and the finger. (**c**) Change in the sensor capacitance before and after finger touching.

It is worth noting that the high-resolution capacitive sensor has a different structure compared with the previous type of flexible capacitive sensor. Figure 2 clearly shows that it consists of two layers of row and column electrodes and a dielectric layer. Notably, there is no protective layer on the outer surfaces of the two electrode layers, implying that the electrodes of this sensor are exposed. As depicted in Figure 5a,b, when the finger does not touch the sensor, the dielectric material between the Rx and Tx electrodes consists of the photopolymer and air. However, when the finger touches the sensor’s top electrode directly, without a protective layer, the air in the dielectric material between the two electrodes is replaced by the finger, which has a different dielectric constant.

Although a portion of the electric field lines passing through the finger is attenuated, the overall capacitance value between the Rx and Tx electrodes still increases [17]. The graph in Figure 5c presents the variation in capacitance values throughout the process. In the initial state of the sensor, the average capacitance value between the two electrodes was approximately 9.17 pF. When the finger touched the electrode, there was a sudden increase in the capacitance value, which stabilized at an average value of approximately 264.89 pF, with fluctuations of around 20 pF.

**Figure 6:**
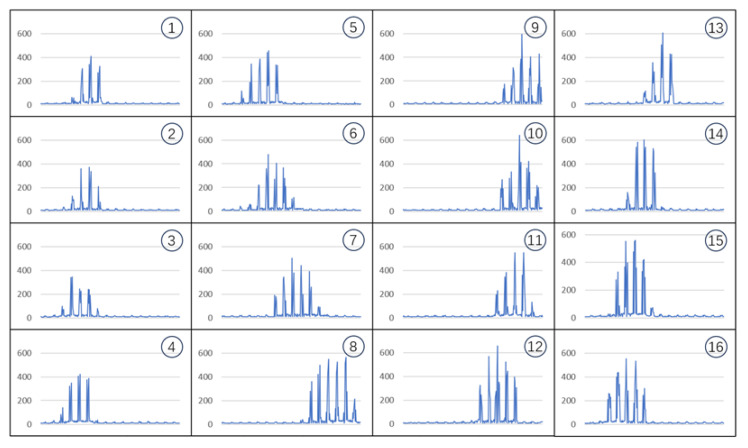
Sequence of capacitance values corresponding to the number “0” trajectory.

The sliding trajectory image is obtained by transforming a sequence of capacitance values measured over a certain period. In the experiment, the time for the finger sliding touch input was set to be the duration of 16 data frames, meaning that a single sample of the capacitance value trajectory was acquired through 16 iterations of system measurement. Figure 6 shows a sample of the capacitance value trajectory after pre-processing, which was drawn using a finger on the surface of a micro-capacitive array sensor to form the number “0”. This sample consisted of temporal data across 16 channels, with the four columns of images presented in order from top to bottom and left to right, representing the waveform plots of the first to sixteenth sets of the capacitance value sequences.

A complete set of capacitance value sequences was measured by a 16 × 16 micro-capacitive array that contained 256 data points, where every consecutive 16 data points corresponded to one array column. Thus, the 1st to 16th data points, the 27th to 32nd data points, …, and the 241st to 256th data points in the capacitance value sequence represented the capacitance values of the 1st, 2nd, …, and 16th columns of the sensor, respectively. On the waveform diagrams of the capacitance value sequences in Figure 6, the stable low-capacitance values represent areas not touched by the finger, while the peaks of high capacitance values indicate areas touched by the finger.

When the finger drew the number “0” on the surface of the micro-capacitive array sensor, it started at the upper-left corner of the “0”. It moved counterclockwise along its shape, and eventually returned to the starting position. As shown in Figure 6, the clusters of high-capacitance value peaks initially shifted slightly to the left, then moved to the right until reaching the end, and finally shifted back to the left until they roughly coincided with the starting position. Similarly, the trajectories for drawing the numbers from “1” to “9” followed the same pattern, and the shapes of the resulting capacitance value sequences exhibited similar characteristics.

### 4.3. Trajectory Visualization

**Figure 7:**
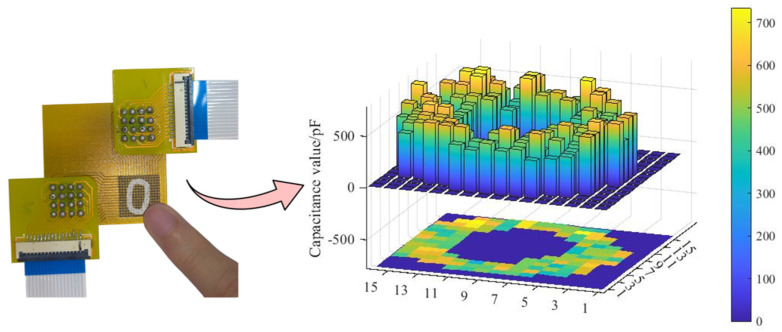
Sliding motion with the index finger and the visualized trajectory of the number “0”.

Therefore, it was feasible to set a threshold to determine touch events on the sensor surface. When the measured capacitance value exceeded this threshold, it could be identified as a touch event. Conversely, values below the threshold were considered as interference and were filtered out. Detecting a sliding touch input is crucial for a tactile sensor designed for human–computer interaction applications [18]. Figure 7 displays the captured trajectory plot and capacitance value mapping when the finger slid within the sensor’s sensing area to trace the shape of the number “0”. It can be observed that the capacitance values in the non-touched regions were disregarded, whereas the capacitance values in the touched region were retained and contributed to the formation of the touching trajectory.

The capacitance value matrix can be plotted as heatmaps to facilitate a more intuitive visualization of the capacitance value distribution and enable better identification of the touch points on the capacitive sensor. These heatmaps provide a clear visual representation of the capacitance values recorded by the sensor, helping to identify the touch points more effectively. The detailed steps involved in generating the heatmaps are as follows:

Step 1: Interpolation

Interpolation is applied to the capacitance value data to enhance the visual clarity of the touch point images and provide more accurate touch point positions. This study used bilinear interpolation to scale up the original 16×16 capacitance value data matrix to a larger size of 160 × 160. Bilinear interpolation increases the resolution of the touch point images, making it easier to discern the exact locations of the touches on the sensor.

Step 2: Threshold segmentation

Threshold segmentation is performed to mitigate the interference caused by potential noise or outliers during the data collection process. A threshold is established to extract the touch points. Experimental findings show that setting the threshold as $(C_{max}+C_{avg})/2$ yielded the most suitable results, where $C_{max}$ represents the maximum value and $C_{avg}$ represents the average value of a particular set of capacitance value data. Following the thresholding procedure, only the capacitance value data corresponding to the touch points are retained. This step helps filter background noise and focus on the actual touch events.

Step 3: Frame merging

Each set of capacitance value data obtained during each iteration (16 × 16) is called a data frame. The touch trajectory can be obtained by merging multiple thresholded data frames and taking the union of all touch regions. This step combines the touch points over time to create a continuous path, allowing us to track the movement of the touch on the sensor.

Step 4: Display of heatmaps

In this heatmap, the capacitance values are depicted using a color scale that transitions through red, yellow, green, and blue, indicating a decreasing order of capacitance values. This color scale provides a clear visual representation of the touch intensity, with deep red indicating the highest capacitance values and deep blue indicating the lowest.

**Figure 8:**
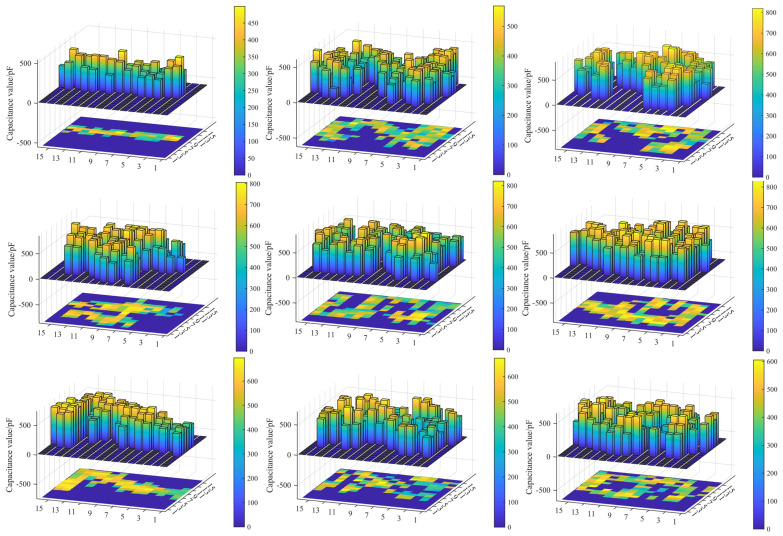
Visualized trajectories from numbers “1” to “9”.

Examples of the heatmaps from the number “0” to “9” can be seen in Figure 8. These heatmaps clearly visualize the touch patterns associated with each number. By comparing the heatmaps, it becomes evident that the shape of each number can be easily discerned.

## 5. Experiment of Finger-Sliding Motion Recognition Based on ACGAN

An experimental evaluation was performed to assess the accuracy of the touch detection of the capacitive tactile sensor. The participants were instructed to use their finger’s tip to slide at a constant and slow pace on the surface of the capacitive sensor, generating the trajectories of the numbers from ”0” to “9”. Simultaneously, the generated trajectory heatmaps were observed on the computer. Each number was repeated 50 times, and attempts were made to ensure consistent positions for each sliding motion. To facilitate the model’s training, a deep learning training environment was set up, with specific configurations detailed in Table 1. We constructed a model for the visualization data of the finger touch trajectory and conducted experiments accordingly.

For machines to respond appropriately based on control signals, they must be capable of “understanding” human instructions, which involves accurate recognition of different touch inputs and generating corresponding output actions accordingly. Presently, a diverse range of algorithms are used for image classification. However, unlike typical image recognition tasks, this research employed a relatively small dataset with many image categories. Employing conventional machine learning classification algorithms in such scenarios may lead to overfitting concerns. Therefore, data augmentation is necessary to expand the dataset’s scale, and in this context, Generative Adversarial Networks (GANs) offer a promising choice [19].

A GAN consists of two main components: a generator and a discriminator. The generator is trained to understand the underlying probability distribution of the dataset, allowing it to produce new images. A well-trained generator can seamlessly integrate its generated images into the original dataset, effectively expanding its size. In a traditional GAN setup, the discriminator can only evaluate the probability distribution of images concerning the original dataset. However, the discriminator can be equipped with image classification capabilities through appropriate adjustments. The field of GAN research has produced numerous variations, and for our classification task involving trajectory images, the ACGAN (Auxiliary Classifier Generative Adversarial Network) was utilized.

### 5.1. ACGAN

**Figure 9:**
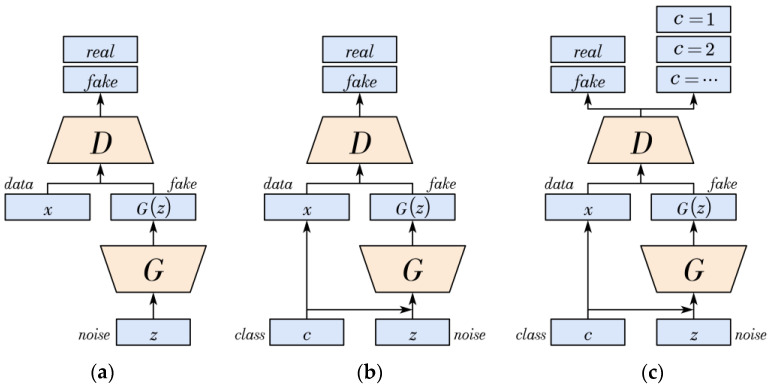
The training processes of the (**a**) GAN, (**b**) CGAN, and (**c**) ACGAN.

The ACGAN is a semi-supervised learning approach proposed by Odena et al. in 2016 [20], building upon the foundation of the original GAN. The original GAN functions relatively simply, with its generator receiving noise data to produce counterfeit images, while the discriminator assesses the authenticity of the images received. The training process of the original GAN is guided by the loss Equation (2), where G and D represent the generator and discriminator, respectively. x denotes real images, and z represents random noise. The objective of the generator is to minimize this equation, while the discriminator aims to maximize it, forming a binary minimax game. The training process of the original GAN is illustrated in Figure 9a.

(2) $$\underset{G}{\min}\underset{D}{\max}V\left(D,G\right)=E_{x\sim p_{data}(x)}\left[logD\left(x\right)\right]+E_{z\sim p_{z}(z)}\left[\log\;(1-D(G\left(z\right)))\right]$$

Due to the limitation of the original GAN in controlling image generation and classifying image categories, the CGAN (Conditional Generative Adversarial Network) was introduced, which utilizes class labels as auxiliary information to guide the image generation process [21]. As a result, the generator can produce specific images based on specified labels. The loss function of the CGAN is represented by Equation (3), where c denotes the image class label. Figure 9b illustrates the training process of the CGAN.

(3) $$\underset{G}{\min}\underset{D}{\max}V\left(D,G\right)=E_{x\sim p_{data}(x)}\left[logD\left(x|c\right)\right]+E_{z\sim p_{z}(z)}\left[\log\;(1-D(G(z|c)))\right]$$

The ACGAN (Auxiliary Classifier Generative Adversarial Network) builds upon the CGAN by introducing an additional improvement, as illustrated in Figure 9c. It incorporates an auxiliary classifier into the discriminator, enabling the discriminator to simultaneously classify the authenticity of images and their corresponding class labels. In contrast to traditional GAN algorithms, the ACGAN’s generator (G) takes random noise and image class labels as inputs. Conversely, the discriminator (D) gives both a probability distribution over sources and a probability distribution over the class labels. The objective function has two parts: the log-likelihood of the correct source, $L_{s}$, and the log-likelihood of the correct class, $L_{c}$, as shown in Equations (4) and (5).

(4) $$L_{s}=E\left[logP\left(S=real|X_{real}\right)\right]+E[logP(S=fake|X_{fake})]$$

(5) $$L_{c}=E\left[logP\left(C=c|X_{real}\right)\right]+E[logP(C=c|X_{fake})]$$

The training objective of the ACGAN is achieved by minimizing two loss functions: the generator loss and the discriminator loss. The generator loss consists of two components: first, the adversarial loss, which encourages the generator to produce realistic image samples that make it difficult for the discriminator to distinguish between real and generated images; second, the classification loss, which encourages the generator to produce images that match the specified labels, enabling the discriminator to accurately classify them. In this case, the generator aims to maximize $L_{s}+L_{c}$. As for the discriminator loss, it also comprises two parts: first, the adversarial loss, which ensures that the discriminator can accurately discriminate between real and generated images; second, the classification loss, which ensures that the discriminator can accurately identify the class label of the input images. The objective of the discriminator is to maximize $L_{s}-L_{c}$.

During training, the generator and discriminator are updated alternately, optimizing their respective loss functions to refine the model parameters. Moreover, with the introduction of the conditional vector, an ACGAN gains the ability to control the class of generated images, which significantly enhances its flexibility and controllability during sample generation.

The ACGAN’s inception has provided an effective solution to the shortcomings of traditional GANs in unsupervised learning and sample generation control, overcoming some of the limitations they faced. First, the class control feature of the ACGAN empowers the model to generate images of specific categories based on designated conditions, enhancing its adaptability. Moreover, ACGAN leverages generated image samples for data augmentation, which contributes to the model’s improved generalization ability.

### 5.2. Datasets

The dataset consisted of 500 trajectory images. Initially, one-fifth of the dataset was chosen as the validation set. The remaining data were then split into the training and test sets in a 4:1 ratio. As a result, the training set contained 320 images, the test set contained 80 images, and the validation set contained 100 images.

### 5.3. Model Construction

**Figure 10:**
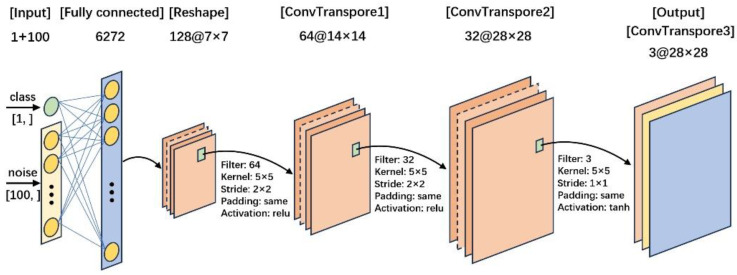
The generator model of the ACGAN.

**Figure 11:**
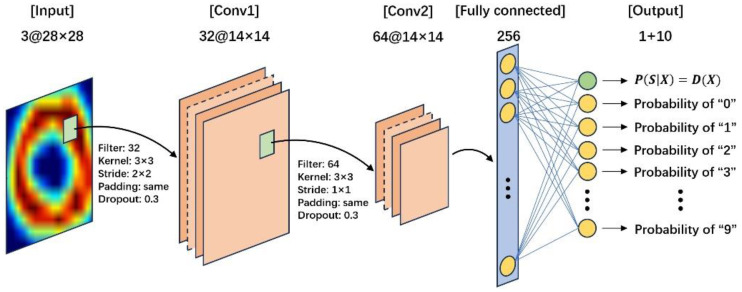
The discriminator model of the ACGAN.

In this experiment, the generator and discriminator models of the ACGAN are depicted in Figure 10 and Figure 11, respectively. The input to the generator model consisted of a noise vector with a length of 100 and a class label with a length of 1. After passing through a fully connected layer, it was reshaped into a tensor of size 7 × 7 × 128. Subsequently, it passed through three transposed convolutional layers to obtain a fake image output of size 28 × 28 × 3. The activation functions used in the first two deconvolutional layers were relu functions. To normalize the generator’s output to the range [−1, 1] for training the discriminator in the next step, the activation function used in the last deconvolutional layer was tanh.

The input to the discriminator model was the image of size 28 × 28 × 3, which could be sourced from either the training set or the generator model’s output. After passing through two convolutional layers, it was connected to a fully connected layer with 256 nodes. Finally, it was further connected to an output layer with 11 nodes. The first node in the output layer gave a probability distribution over sources, while the remaining ten nodes gave the probability distribution over classes of numbers “0” to “9”.

### 5.4. Results

**Figure 12:**
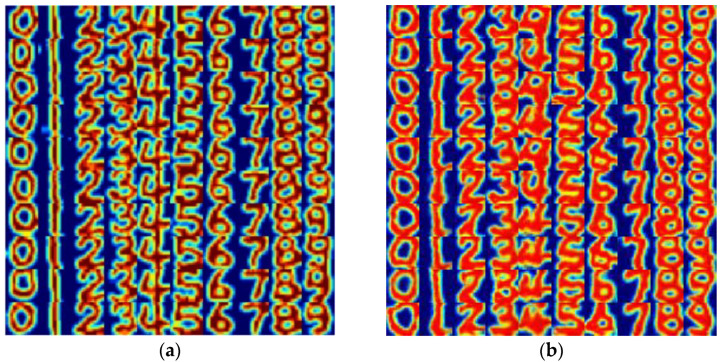
The trajectory heatmaps of the numbers “0–9”: (**a**) trajectory heatmaps obtained by drawing numbers on the capacitive sensor using a finger; (**b**) trajectory heatmaps generated using a GAN model’s generator.

As a result of the experimental procedure, a total of 500 trajectory heatmaps were obtained. These heatmaps provided a visual representation of the touch trajectories recorded by the micro-capacitive sensor. Figure 12a displays a selection of these heatmaps, showcasing the typical patterns observed during this study. Additionally, the trajectory heatmaps generated using the Generative Adversarial Network (GAN) model’s generator can be seen in Figure 12b. The use of a GAN model allowed for the creation of synthetic heatmaps that closely mimicked the touch trajectories captured by the sensor.

Upon meticulous comparison and detailed observation of both sets of heatmaps, it became apparent that the shape of each number (i.e., finger) could be easily discerned within the visual representations. The precise definition of the number shapes in the real and generated heatmaps indicated that the system could reliably distinguish between the different numbers and accurately reproduced the touch points on the sensor’s surface. Furthermore, there was a tiny variation between the shapes that corresponded to the same number across different trials. This consistency suggested that the micro-capacitive sensor system exhibited excellent repeatability and precision in recording the touch interactions. This finding is crucial for confirming the system’s capability to accurately reproduce the touch points, which is essential for applications requiring precise touch recognition and tracking, such as in advanced human–computer interaction interfaces or biomedical research.

**Figure 13:**
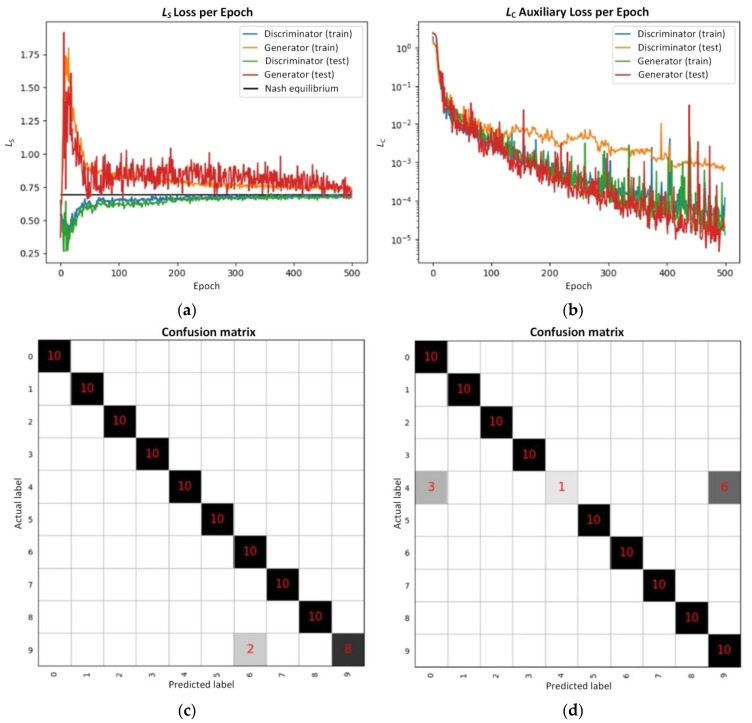
The (**a**) loss and (**b**) auxiliary loss of the model, as well as the confusion matrix for the discriminator model on the (**c**) validation set and (**d**) fake image set.

Following 500 training epochs, the loss curves for the generator and discriminator models are depicted in Figure 13a. It can be observed from the graph that both models reached stability and converged around the 300th epoch, ultimately attaining a satisfactory Nash equilibrium by approximately the 500th epoch. Figure 13b presents the auxiliary loss curve, which is associated with the discriminator’s accurate classification of image categories. Throughout the training process, both models aimed to minimize this loss. After 500 epochs, this loss had been reduced to 10^−4^, approaching 10^−5^. Hence, it can be inferred that the constructed model achieved excellent training performance.

To evaluate the performance of the generator, noise vectors, along with the “0–9” class labels, were input into the well-trained generator model to generate 10 images for each number, as demonstrated in Figure 12b. From these images, it can be observed that the number images appear pretty realistic and are challenging to distinguish from the images in the training set with the naked eye. Although a few generated samples may exhibit slight variations in shape compared with their corresponding numbers, their categories can still be visually discerned. Overall, it can be inferred that the generator effectively reproduced the probability distribution of the images in the dataset.

The discriminator model achieved an accuracy of 98% on the validation set, as evidenced by the confusion matrix in Figure 13c. However, the model made two misclassifications by mistakenly identifying “9” as “6”. Subsequently, when the generated number images from the generator were consolidated into a new dataset, labeled as the “fake image set”, the model exhibited a slightly lower accuracy of 91% on this dataset. The confusion matrix for this dataset is illustrated in Figure 13d. Notably, the model displayed low recognition performance for “4”, while excelling in recognizing the other images.

## 6. Conclusions

This paper introduces the design and fabrication of a novel type of 16 × 16 capacitive tactile sensor using inkjet deposition printing technology, with a sensing area of 40 mm × 40 mm and a higher resolution of 32.7 dpi. These sensors are intended for wearable applications, offering excellent thinness and lightweight properties. The changes in the capacitance distribution of the capacitive sensors could be accurately captured with a scanning rate of 100 Hz, enabling precise touch point detection. A series of experiments were devised to validate the precise tactile detection of the sensor, and it was achieved by generating touching trajectories on the sensor surface. Furthermore, to assess the feasibility of controlling various functions by drawing patterns on the sensor surface, the touch trajectories of the numbers from “0” to “9” were subjected to recognition using the ACGAN algorithm, which resulted in an accuracy of 98% on the validation set.

## Figures and Tables

**Table 1:** Configuration of the training environment.

Components	Specifications
Operating system	Ubuntu 22.0.4
Integrated development environment (IDE)	PyCharm 2022.1.1
Programming language	Python 3.7
Deep learning framework	PyTorch
CPU	Intel i9-13900K
Memory	128 GB
GPU	NVIDIA GeForce RTX 4090

## Data Availability

The data presented in this study are available on request from the corresponding author.
